# Unveiling the hidden dynamics: patellar support tissue alterations in post-stroke rehabilitation

**DOI:** 10.3389/fresc.2026.1838086

**Published:** 2026-08-06

**Authors:** Yongqian Han, Shaofang Li, Juchuan Dong, Fuhou Zhang, Yongjun Gao, Lihua Jin

**Affiliations:** 1 Department of Rehabilitation Medicine, Second Affiliated Hospital of Kunming Medical University, Kunming, Yunnan, China; 2 Department of Neurosurgery, Second Affiliated Hospital of Kunming Medical University, Kunming, Yunnan, China

**Keywords:** biomechanical, body balance, patella, patellar support, post-stroke rehabilitation

## Abstract

**Background:**

This study sought to evaluate the thickness of supportive bands and ligaments surrounding the patella in post-stroke patients, with the objective of informing tailored rehabilitation interventions. This is necessitated by the ambiguous effects of tissue alterations on pain and joint injury.

**Methods:**

We assessed first-time stroke patients aged 30–80 years within one year post-onset using ultrasound. The thickness of the patellar tendon, quadriceps tendon, and medial and lateral patellofemoral ligaments was measured on both affected and unaffected sides. We also evaluated the differences in thickness (horizontal and vertical power) across patients with varying disease durations.

**Results:**

Significant differences in horizontal power were found between sides, diminishing on the unaffected side with longer disease duration. Vertical power varied on the affected side depending on whether the disease duration was above or below 60 days. The mean power center shifted medially and laterally on the affected and unaffected sides, respectively, within the first 60 days, and further shifted downwards and laterally in patients beyond 60 days.

**Conclusions:**

Support tissue distribution around the patella differs between sides in hemiplegic patients, with changes becoming more pronounced over time. Rehabilitation should include targeted muscle training to prevent knee injuries and enhance recovery.

## Introduction

Patients with hemiplegia encounter a range of gait disturbances, including asymmetry, reduced step length, decreased speed, and increased variability in the spatiotemporal parameters of gait (1). These issues can result in biomechanical abnormalities within the musculoskeletal system on the paretic side, with the paretic knee joint facing specific challenges, such as potential overuse, increased loading, and periarticular calcification (2). As a major weight-bearing joint, post-stroke knee pain can substantially affect rehabilitation. Given that most patients prioritize the recovery of physical function over cognitive or emotional function, particularly in China (3), understanding and specifically preventing the occurrence and exacerbation of knee joint abnormalities in these patients is essential. Hemiparetic patients typically exhibit abnormal knee joint alignment during both static and dynamic weight-bearing activities, attributable to alterations in the line of force. This misalignment can result in elevated tibiofemoral contact forces, thereby contributing to joint wear, particularly at the patellofemoral and tibiofemoral interfaces (4). Notably, the wear induced by these increased contact forces may precipitate knee pain on both the paretic and non-paretic sides (5). Consequently, the aberrant gait patterns observed in stroke patients may cause a shift in the center of pressure, exacerbating joint damage.

Osteoarthritis is recognized as a comorbidity in individuals who have experienced a stroke (6). Post-stroke patients exhibit comparable levels of soft tissue joint degeneration in both the paretic and non-paretic limbs (5). This concomitant injury arises not only from the alteration of the mechanical axis due to asymmetrical weight-bearing in the lower limbs (7), but also from postural sway (8), which subsequently intensifies the wear on the unaffected side (9). Furthermore, this asymmetry in weight-bearing has a direct impact on functional walking performance, thereby elevating the risk of falls in real-world settings (8, 10). Although there are documented instances of cartilage involvement in both the paretic and non-paretic limbs following a stroke (11), limited research has been conducted on alterations in patellar positioning post-stroke. The position and movement of the patella are significantly influenced by biomechanical changes within the knee joint, as variations in pressure distribution can result in modifications to the movement trajectory and positioning of the patella. Patellar alignment and adjustment are critical factors influencing the onset and progression of knee osteoarthritis. The stabilization of the patella is primarily facilitated by the medial and lateral patellofemoral ligaments, which include the medial patellofemoral ligament, medial patellotibial ligament, lateral patellofemoral ligament, and lateral patellotibial ligament. These ligaments collectively ensure medial and lateral stability. Post-stroke, these structures frequently experience changes, particularly atrophy, which can lead to alterations in patellar positioning. Such displacement of the patella may result in uneven pressure distribution within the patellofemoral joint, potentially causing or exacerbating structural damage to the joint (12).

Consequently, we propose the hypothesis that in patients with post-stroke hemiplegia, neuromuscular dysfunction may result in differential alterations in the bilateral patellar support structures, potentially correlating with the duration of the disease. To explore this hypothesis, we conducted a comparative analysis of the thickness of the primary supporting bands on the medial and lateral aspects of the patella on both the unaffected and affected sides in stroke patients. Given the cross-sectional nature of this study, our analysis focused on associations between groups with different disease durations rather than longitudinal changes within individuals.

## Methods

### Sample size estimation

The sample size for this study was determined by referencing the sample sizes reported in prior studies employing lower limb characteristics in stroke patients (10). An online sample size calculator (https://sample-size.net/) was utilized, with the difference in muscle thickness between the unaffected and affected sides as the indicator. Assuming an effect size of 0.7 and a statistical power of 1 − *β* = 0.80, the number of required participants was calculated. A total of 72 samples were needed.

### Study design

This single-center, cross-sectional investigation was conducted at one of the largest rehabilitation centers in the one of the provinces on southwestern region of China from March to June 2025.

Prior to inclusion, all patients and/or their guardians were informed of the study objectives and provided written informed consent. This study adhered to the ethical principles outlined in the Declaration of Helsinki.

### Participants

The inclusion criteria were individuals aged 30–80 years with a first-time diagnosis of ischemic or hemorrhagic stroke within the past year; those who could stand with assistance and possess a standing balance of at least grade 1; those with muscle strength of the unaffected lower limb, evaluated using the Manual Muscle Test (MMT), of at least grade 4; those whose clinical conditions were stable with no history of diabetes or other consumptive diseases; and those with no history of falls or fractures prior to stroke and no clear history of osteoarthritis. To minimize measurement bias due to tension issues, spasticity in the lower limbs, assessed using the Modified Ashworth Scale (13), was required to be less than grade 2. Furthermore, participants had no apparent deformities in the lower limb joints.

### Measurements of tissue thickness and grayscale

The acquisition and measurement of ultrasound data were conducted by two rehabilitation physicians (Y.Q.H., S.F.L.) certified in musculoskeletal ultrasound, whereas the data were subsequently reviewed by an additional rehabilitation physician (Y.M.L.) possessing over a decade of experience in ultrasound practice. Prior to formal testing, an assessment of measurement reliability was conducted for muscle thickness and grayscale in a cohort of 10 patients, with evaluations performed collaboratively by two physicians (Y.Q.H., S.F.L.). The intra-class correlation coefficient (ICC) within the group ranged from 0.86 to 0.93, while the inter-class ICC was determined to be 0.9. These findings suggest that the measurements demonstrate a high degree of reliability. A 6–15 MHz linear-array transducer equipped with M-mode ultrasonography (Sonimage HIS; Konica Minolta, Tokyo, Japan) was employed. Participants were instructed to assume a supine position, ensuring that their lower limbs were naturally extended and relaxed to minimize movement or exertion, which could introduce bias. The ultrasound probe was positioned parallel to the superior border of the patella, 1 cm from the patella, to measure the quadriceps tendon thickness, and parallel to the inferior border, 1 cm from the patella, to assess the patellar tendon thickness. For the medial and lateral retinacula, the probe was oriented perpendicular to the patella, and measurements were taken 1 cm medial and lateral to the patella, respectively. To enhance accuracy, measurements were recorded at the center of the probe and on either side, and the mean value was calculated. The measurement method of tissue thickness refers to the previously published literature (14, 15). The methodology employed for measuring grayscale aligns with the protocol established in our prior study (16). Initially, the region of interest within the tissue is delineated, and subsequently, the measurement is conducted utilizing ImageJ software.

### Outcomes

In light of the influence of tissue structures on patellar coordination, we conducted simultaneous measurements of the thicknesses of the quadriceps tendon, patellar tendon, medial retinaculum, and lateral retinaculum. The disparity in thickness between the quadriceps and patellar tendons was defined as the vertical power, while the difference between the lateral and medial retinacula was termed the horizontal power. These indices were introduced as exploratory measures aimed at identifying potential asymmetries in the distribution of supportive forces surrounding the patella. The rationale for employing thickness differences as a surrogate for biomechanical imbalance is grounded in the well-documented correlation between soft tissue thickness and its load-bearing capacity (10, 17, 18). It is important to acknowledge, however, that these indices have not yet been validated as standardized biomechanical metrics, and their clinical relevance remains to be determined. Consequently, they are presented as preliminary, hypothesis-generating parameters in the present study.

To evaluate the alterations in muscle strength between the unaffected and affected sides following a stroke, we calculated the delta value by subtracting the thickness measurements of the tendons or retinacula on the affected side from those on the unaffected side. These delta values were then compared across different disease durations to elucidate the changes in the tissues surrounding the patella as the condition advanced. Given the substantial correlation between muscle thickness and both albumin and cholesterol levels (19, 20), along with the intrinsic link between muscle thickness and functional independence (21), we also incorporated the albumin (ALB), total Cholesterol (TCHOL), and functional Independence Measure (FIM, motor and total score) into our study for comprehensive analysis.

### Statistical analysis

Owing to the non-normal distribution of many data points, the Wilcoxon paired *t*-test was employed to compare tissue thickness, vertical imbalance, and horizontal imbalance between the affected and normal sides. The Mann–Whitney *U* test was used to compare differences between patients with an onset duration of more or less than 60 days. The determination of the demarcation point in this study primarily arises from an examination of the thickness and grayscale of the peripatellar retinaculum, contingent upon the disease progression prior to the comprehensive data analysis (Supplementary Data 1). In evaluating the tissue characteristics at intervals of 30, 60, and 90 days, only the delta value of the patients' tissues at the 60-day mark demonstrated a significant difference. Consequently, the 60-day interval was selected as the demarcation point for subsequent analyses. Spearman correlation analysis was used for correlation analysis, and data analysis was performed using SPSS 30.0 software (IBM Corp., Armonk, NY, USA). GraphPad Prism 8.0 (GraphPad Software, San Diego, CA) was used to plot graphs, with horizontal imbalance on the *x*-axis and vertical imbalance on the *y*-axis. Each point in the figure represents the horizontal difference values between the medial and lateral supporting bands and the vertical difference values between the quadriceps tendon and patellar tendon for each patient, with the highlighted points indicating the average values of these measurements.

## Results

### Demographic and clinical characteristics

We included a cohort of 72 patients with post-stroke hemiplegia; however, two participants were excluded from the analysis due to suboptimal ultrasound data. In total of 70 patients, with a mean age of 55.71 ± 13.46 years and a mean disease duration of 67.19 ± 54.6 days. The cohort was evenly divided into 35 patients with left hemiplegia and 35 with right hemiplegia. Of these patients, 36 experienced ischemic stroke and 34 had hemorrhagic stroke. Five patients reported unilateral knee pain, seven reported bilateral knee pain, and 58 reported no history of knee pain (Table 1).

**Table 1:** Patients’ demographic characteristic.

Item	*n*
Age (year)	57 (30, 80)
Weight (kg)	69 (40, 90)
Duration (day)	50 (7, 295)
Sex	
Male	54
Female	16
Affected side	
Right	35
Lift	35
Type of stroke	
Ischemic	36
Hemorrhage	34
History of knee pain	
None	58
Yes	5
Both knee	7
FIM motor	20 (13, 87)
FIM total	35 (18, 104)
ALB	37.5 (29.2, 47.9)
TCHOL	3.47 (2.02, 5.59)

ALB, albumin; FIM, functional independence measure; TCHOL, total cholesterol.

**Figure 1:**
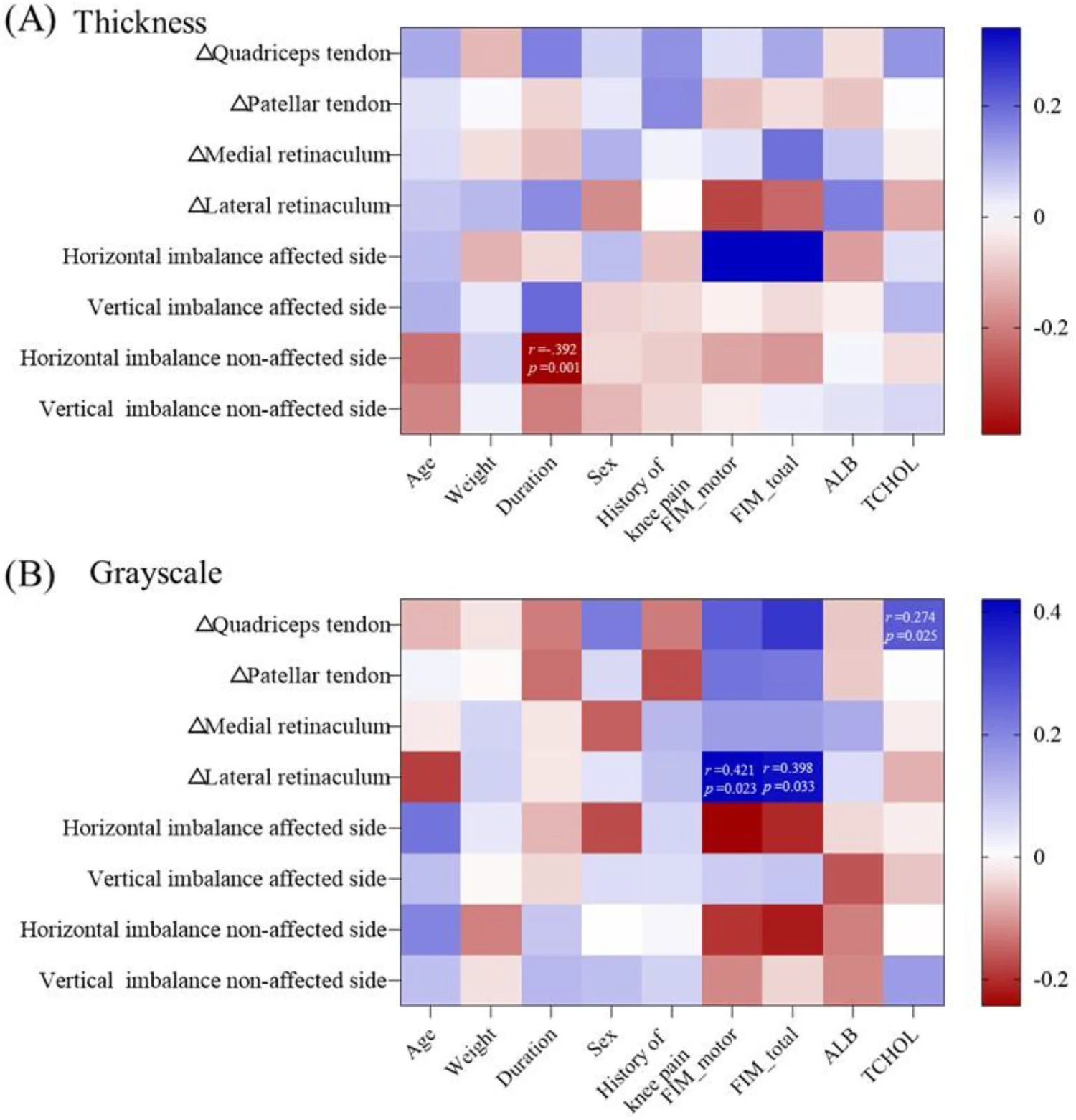
Influence of demographic characteristics and the thickness and grayscale of tissues. **(A)** Correlation between tissues thickness and demographic characteristics; **(B)** Correlation between tissues grayscale and demographic characteristics. △, delta value, non-affected side minus affected side; ALB, albumin; FIM, functional independence measure; horizontal imbalance, the delta value of the lateral minus medial patellofemoral ligament thicknesses; TCHOL, total cholesterol; vertical imbalance, the delta value of the quadriceps minus patellar tendon thicknesses.

A comparative analysis of tissue thickness, horizontal imbalance, and vertical imbalance between the unaffected and affected sides revealed a statistically significant difference in horizontal imbalance (*p* = 0.047). In the context of grayscale analysis, the affected side exhibited notable differences compared to the non-affected side, particularly in the grayscale measurements of the quadriceps tendon (*p* = 0.008) and the medial support structure (*p* = 0.033). Specifically, the quadriceps tendon displayed higher grayscale values on the non-affected side, whereas the medial support structure exhibited higher grayscale values on the affected side (Table 2). Furthermore, correlation analysis indicated that, regarding thickness, horizontal power on the unaffected side showed a weak negative correlation with disease duration (*r* = −0.392, *p* = 0.001). In terms of grayscale, the delta value of the quadriceps tendon was weakly positively correlated with TCHOL levels (*r* = 0.274, *p* = 0.025), while the delta value of the lateral support structure demonstrated a weak positive correlation with the FIM total score (*r* = 0.398, *p* = 0.033) and a moderate positive correlation with the FIM motor score (*r* = 0.421, *p* = 0.023) (Figure 1).

**Table 2:** Thickness and grayscale of tissues on the affected and non-affected sides.

Item	Affected side	Non-affected side	*p* value
Thickness			
Quadriceps tendon	6.07 (3, 7.97)	6.27 (2.87, 8.33)	0.615
Patellar tendon	3.19 (1.83, 12.83)	3.15 (2.03, 5.1)	0.733
Medial retinaculum	2.54 (1.5, 11.4)	2.4 (1.43, 4.13)	0.167
Lateral retinaculum	2.73 (1.77, 6.77)	2.74 (1.5, 4.6)	0.367
Horizontal imbalance	0.1 (−6.93, 4.87)	0.32 (−1.2, 1.97)	**0** **.** **047** *
Vertical imbalance	2.52 (−6.16, 5.6)	2.75 (−0.7, 5.1)	0.488
Grayscale			
Quadriceps tendon	50.219 (23.821, 91.025)	58.7 (28.034, 97.244)	**0** **.** **008** *
Patellar tendon	50.552 (23.378, 114.26)	49.271 (22.015, 577.199)	0.639
Medial retinaculum	91.715 (44.682, 128.861)	86.511 (47.057, 114.487)	**0** **.** **033** *
Lateral retinaculum	77.039 (33.785, 117.879)	78.063 (50.268, 111.368)	0.915
Horizontal imbalance	−11.742 (−63.453, 64.385)	1.079 (−36.111, 41.777)	0.177
Vertical imbalance	−9.063 (−51.989, 24.857)	8.658 (−505.107, 63.158)	0.147

*A comparison between the affected side and the non-affected side, *P* < 0.05.

To elucidate the temporal changes in the tissues surrounding the patella, we stratified the patients into two cohorts based on disease duration: those with a disease duration exceeding 60 days and those with a disease duration of 60 days or less. Subsequently, we performed a comparative analysis of the general conditions, tissue thickness, and grayscale values between the two patient cohorts. The analysis revealed a statistically significant difference in vertical imbalance on the affected side concerning tissue thickness between the groups (*p* = 0.013). Additionally, the delta value of the patellar tendon grayscale demonstrated a statistically significant difference between the groups with two disease durations (*p* = 0.040) (Table 3).

**Table 3:** Difference of the thickness of tissues between the different onset durations after stroke.

Item	≤60 day	>60 days	*p* value
Age	56 (31, 80)	59 (30, 80)	0.958
Weight	68 (40, 90)	70 (40, 81)	0.967
Duration	33 (7, 59)	93 (61, 295)	0.000
Sex			
Male	27	27	0.079
Female	12	4	
History of knee pain			
None	31	27	0.387
Yes	3	2	
Both knee	5	2	
FIM motor	21.5 (13, 62)	19 (13, 87)	0.721
FIM total	40 (21, 95)	31 (18, 104)	0.492
ALB	37.7 (32.6, 47)	37.4 (29.2, 47.9)	0.603
TCHOL	3.62 (2.23, 5.59)	3.28 (2.02, 5.26)	0.063
Tissue thickness			
△Quadriceps tendon	−0.06 (−3.73, 3.26)	0.2 (−1.77, 3.43)	0.497
△Patellar tendon	0.07 (−1.33, 1.9)	−0.1 (−9.16, 0.76)	0.344
△Medial retinaculum	−0.06 (−9.1, 2.06)	−0.04 (−2.17, 0.73)	0.981
△Lateral retinaculum	0 (−4.37, 1.43)	0.27 (−1.04, 1.23)	0.129
Horizontal imbalance Affected side	0.1 (−6.93, 4.87)	0.14 (−1.7, 1.03)	0.781
Horizontal imbalance Non-affected side	0.2 (−1.17, 1.04)	0.56 (−1.2, 1.97)	0.073
Vertical imbalance Affected side	3.06 (0.47, 5.6)	2.2 (−6.16, 4.87)	**0** **.** **013** *
Vertical imbalance Non-affected side	2.94 (−0.7, 5.1)	2.56 (0.64, 5.1)	0.187
Tissue grayscale			
△Quadriceps tendon	6.624 (−38.268, 47.025)	5.519 (−42.69, 52.712)	0.615
△Patellar tendon	7.593 (−29.271, 519.714)	−5.298 (−57.377, 391.958)	**0** **.** **040** *
△Medial retinaculum	−4.301 (−37.036, 44.654)	−6.112 (−32.687, 36.849)	0.930
△Lateral retinaculum	4.206 (−53.18, 39.747)	2.196 (−46.03, 49.976)	0.910
Horizontal imbalance Affected side	−12.375 (−47.367, 64.385)	−11.742 (−63.453, 26.541)	0.950
Horizontal imbalance Non-affected side	−8.801 (−37.291, 24.857)	−10.293 (−51.989, 21.718)	0.237
Vertical imbalance Affected side	0.509 (−27.123, 29.936)	1.079 (−36.111, 41.777)	0.365
Vertical imbalance Non-affected side	7.289 (−505.107, 45.966)	12.16 (−405.745, 63.158)	0.458

ALB, albumin; FIM, functional independence measure; horizontal imbalance, the delta value of the lateral minus medial patellofemoral ligament thicknesses; TCHOL, total cholesterol; vertical imbalance, the delta value of the quadriceps minus patellar tendon thicknesses; △ delta value, non-affected side minus affected side.

*A comparison between the disease duration more or less than 60 days, *P* < 0.05.

**Figure 2:**
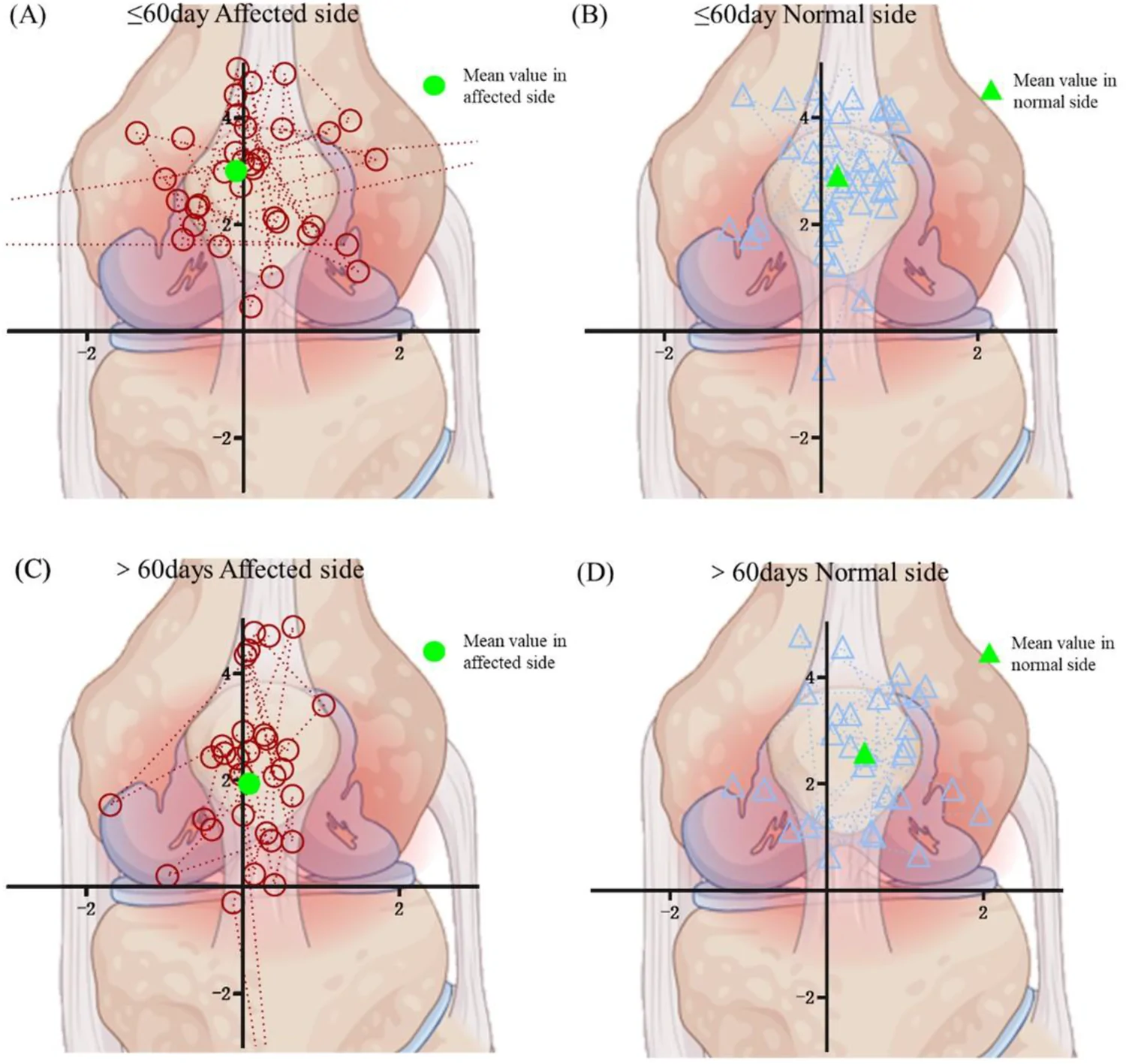
**(A)** Temporal changes in the balance of the peripatellar support in the affected knee joints of patients with a disease duration of less than 60 days; **(B)** Temporal changes in the balance of the peripatellar support in the unaffected knee joints of patients with a disease duration of less than 60 days; **(C)** Temporal changes in the balance of the peripatellar support in the affected knee joints of patients with a disease duration of more than 60 days; **(D)** Temporal changes in the balance of the peripatellar support in the unaffected knee joints of patients with a disease duration of more than 60 days.

To facilitate the comprehension and visualization of these temporal changes, we constructed a graph plotting tissue thicknesses: the thickness of the lateral retinaculum is represented on the positive *x*-axis, thickness of the medial retinaculum on the negative *x*-axis, thickness of the quadriceps tendon on the positive *y*-axis, and thickness of the patellar tendon on the negative *y*-axis. Figure 2 effectively illustrates the delta values of the horizontal imbalance on the *x*-axis and the vertical imbalance on the *y*-axis. Notably, in patients with a disease duration of 60 days or less, the mean tissue thickness on the affected side exhibited an upward and medial shift than unaffected side, whereas that on the unaffected side demonstrated an upward and lateral shift than affected side. In contrast, in patients with a disease duration exceeding 60 days, the mean tissue thickness on the affected side exhibited downward and slight lateral displacement than duration less than 60 days. However, on the unaffected side, the displacement was more pronounced laterally and downward in patients with a disease duration exceeding 60 days compared with those with a disease duration of 60 days or less (Figure 2).

## Discussion

The findings of our study indicate significant variations in tissue thickness and grayscale values of peripatellar structures between the affected and unaffected sides. Specifically, the disparity in tissue thickness within the horizontal plane suggests that the lateral support structures on the unaffected side exhibit greater thickness compared to those on the medial side. This observation is corroborated by the grayscale analysis, which reveals a notably lower grayscale value for the quadriceps tendon on the affected side relative to the unaffected side, while the grayscale value of the medial support structure is more pronounced on the affected side compared to the unaffected side. These modifications may suggest a comprehensive shift in the patellar positioning on the affected side. Furthermore, variations in tissue thickness and grayscale intensity are discernible between patient cohorts with a disease duration of 60 days or less and those with a duration exceeding 60 days. This is particularly notable concerning the vertical thickness imbalance on the affected side and the grayscale discrepancy between the patellar tendons of the unaffected and affected sides. Nonetheless, this remains an indirect inference derived from tissue-level observations and necessitates corroboration through direct imaging-based positioning evidence. In summary, among hemiparetic patients, the patellar alignment in the superior, inferior, medial, and lateral directions may exhibit an outward deviation, progressively shifting outward and upward as the condition advances. In contrast, the patellar alignment on the unaffected limb may initially be closer to the medial side, gradually shifting inward and downward as the condition progresses. It is important to note that this study is unable to draw conclusions about longitudinal progression over time. The inferences presented are based exclusively on inter-group comparisons among patients with varying disease durations, rather than on longitudinal follow-up observations of the same patients.

As patients recovering from stroke progress from the acute phase to the recovery phase, there is a gradual restoration of strength in the muscles surrounding the affected knee. Increased muscle tension during movement, coupled with the contraction of agonist and antagonist muscles, impedes effective separation, abduction, or extension of the knee joint, resulting in abnormal gait patterns (22). Such gait abnormalities in hemiparetic patients may result in heightened dynamic knee joint stiffness, abnormal ground reaction forces, altered joint surface contact, and increased soft tissue wear. While Kong et al. (23) reported that 2% of stroke hemiparetic patients experience knee pain on the non-affected side, other studies have indicated that this prevalence may be as high as 12.5% (5). These findings suggest the presence of biomechanical abnormalities in hemiparetic patients, which may elevate the risk of subsequent injury.

Previous research investigating the biomechanical alterations in the knee joints of hemiparetic patients has predominantly concentrated on variables such as flexion-extension angles, torque, and gait-related parameters, including walking speed, stride length, and joint angles (24–27). Nevertheless, these studies frequently do not adequately capture the progressive changes occurring in the periarticular tissues of both the affected and unaffected sides of the knee joint, particularly within the framework of rehabilitation interventions that emphasize muscle strength and power. Consequently, these indicators merely suggest potential abnormalities and fail to offer comprehensive guidance for training.

Our focus on the patella is due to the fact that the supporting structures surrounding it provide direct insights into alterations in the forces exerted by the extensor muscles (17). As the condition advances and gait abnormalities become more pronounced, these alterations become increasingly evident. For instance, during quiet standing, there is an increased weight-bearing on the unaffected limb, and during dynamic movements, the unaffected limb's contribution to anterior-posterior balance control also rises, while the weight-bearing capacity of the affected limb diminishes (28). These immediate postural adjustments primarily reflect transient states; however, to sustain these postures, muscles and tissues undergo adaptive modifications over time. By assessing these tissues, we can observe the long-term changes that occur following prolonged abnormalities, particularly with respect to alterations in patellar positioning. The observed variations in tissue parameters across different disease-duration cohorts in this study provide valuable insights into the understanding of disease progression. Nonetheless, these differences should not be directly interpreted as indicative of the temporal evolution of the disease in individual patients.

The efficacy of ultrasound in the evaluation of tendons is well-documented. Quantitative analysis of the grayscale in ultrasound imaging allows for a more detailed assessment of tendon quality. While parameters such as tissue thickness and grayscale values do not directly quantify tissue strength, the presence of atrophic tissue and diminished grayscale values may suggest a reduction in tensile strength. This reduction could potentially result in pain or displacement at attachment sites. This consideration is especially pertinent for structures such as the patella, which depends on multidirectional tissue support to maintain its position during knee flexion (29). The medial and lateral supporting bands play a crucial role in ensuring the accurate positioning of the patella (17). However, in patients with stroke, diminished flexion torque in the affected knee may lead to disuse of the lateral retinaculum, resulting in a more pronounced reduction in tissue thickness. Conversely, on the unaffected side, the lateral retinaculum may require greater force to assist to maintain owing to a shift in the center of gravity, leading to an increase in its thickness. In addition to the retinacula, the ligaments, particularly those associated with the medial and lateral vastus muscles, serve as the principal forces supporting the superior displacement of the patella. In the unaffected lower limbs of patients with stroke, increased weight-bearing may augment the force exerted by the lateral vastus muscles, potentially leading to a superior and lateral shift in mean tissue thickness. Conversely, in the affected lower limbs of patients with stroke, the presence of knee vargus and hyperextension may increase the medial load on the knee joint, thereby further reducing the medial cartilage thickness and causing a medial shift in the mean thickness of the affected limb. Furthermore, with disease progression, continued atrophy of the quadriceps femoris, which is the primary source of strength and is accompanied by neural innervation, may result in an inferior shift in the mean value.

To our knowledge, this study represents the first attempt to compare the thickness and grayscale of peripatellar tissues and present the results in a visual manner. It provides a foundation for generating preliminary hypotheses. We systematically plotted the mean thickness of these tissues along the left-right and superior-inferior axes to enhance clarity and present the data in a user-friendly manner. This methodology can serve as an exploratory basis for rehabilitation strategies and provide a theoretical foundation for targeted knee strength training. Post-stroke muscle atrophy, disuse, or overuse may alter the forces exerted by the tissues surrounding the patella, thereby disrupting the originally maintained balanced “arch power.” These changes become more pronounced with increasing disease duration. Therefore, rehabilitation interventions should be tailored to each patient. For example, on the affected side, training should focus on strengthening the lateral muscles and ligaments, whereas on the unaffected side, emphasis should be placed on the medial muscles and ligaments to restore balance and mitigate the offset “arch power.” From a clinical standpoint, it is observed that contemporary rehabilitation assessments for stroke patients primarily emphasize strength grading, range of motion, and gait analysis. There is a notable lack of attention to the structural integrity of supporting soft tissues and the asymmetrical remodeling resulting from strength imbalances, as well as the strength-related discrepancies between the affected and unaffected sides. These factors could potentially inform rehabilitation assessments and clinical decision-making processes, thereby reducing the risk of secondary injuries and facilitating targeted, intensive training.

### Limitations

This study possesses certain limitations, primarily due to its cross-sectional design. While preliminary analyses suggest that alterations in the peripatellar retinaculum may be more pronounced at the 60-day interval, the cross-sectional nature of the study constrains our ability to delineate the precise temporal progression of these changes. Consequently, the proposed rehabilitation strategies should be considered exploratory hypotheses rather than definitive clinical guidelines. Furthermore, the study did not include a healthy control group. Instead, the non-paretic side was used as a within-subject reference, a common approach in stroke research. However, it is important to note that this limb may not accurately represent normal anatomy, as bilateral degenerative changes have been documented in post-stroke patients (11). Ultrasound was initially chosen for assessment due to its convenience and rapidity as a measurement method. Nevertheless, when compared to magnetic resonance imaging, it may present a higher risk of error in the evaluation of tendons and supporting ligaments (30). We assessed the tissue thickness and grayscale rather than strength. Although these parameters are interrelated, their precise causal relationships require further investigation. Prior research has indicated that the medial patellar tilt influences symptoms and structural integrity (31); however, alterations in tissue thickness on the paretic side and their implications for the substantive structure of the patella warrant comprehensive analysis encompassing structural, symptomatic, and osseous changes. Furthermore, our study did not account for anatomical anomalies, e.g., variations in the origin and insertion of the vastus medialis muscle or the positioning of the patella, as our primary objective was to elucidate the changes occurring in the tissues surrounding the patella. Additionally, we did not differentiate between ischemic and hemorrhagic strokes. Given that the recovery processes may differ between these two types of strokes, potentially influencing the characteristics of muscle tissues, future research should address these variables. Lastly, this study included participants aged 30–80 years. In the context of muscle research, this broad age range may have introduced biases related to age-associated degeneration. Future studies should focus on more narrowly defined age groups to achieve more precise assessments.

## Conclusions

In patients with hemiparesis following a stroke, the bilateral lower limbs, particularly the patella, may exhibit distinct directional shifts. On the unaffected side, the patella typically migrates upward and outward, whereas on the affected side, the surrounding supportive structures may induce a gradual inward and downward shift of the patella. Although further investigation is warranted to substantiate this perspective, our study offers novel insights into rehabilitation strategies. It suggests that therapeutic focus should encompass not only the affected side but also the changes observed on the unaffected side. This comprehensive approach may mitigate and prevent excessive abnormal utilization of the knee joint post-stroke, thereby reducing the potential for pain and enhancing the efficacy of rehabilitation interventions.

## Data Availability

The raw data supporting the conclusions of this article will be made available by the authors, without undue reservation.
